# Alarmingly Increased Public Interest in “Chest Pain” During the COVID-19 Pandemic: Insights From Google Trends Analysis

**DOI:** 10.7759/cureus.14292

**Published:** 2021-04-04

**Authors:** Hee Kong Fong, Sandeep Singh, Jilmil S Raina, Vikram B Itare, Violeta Spasova, Rupak Desai

**Affiliations:** 1 Cardiovascular Medicine, University of California Davis Medical Center, Sacramento, USA; 2 Clinical Epidemiology, Biostatistics and Bioinformatics, Academic Medical Center University of Amsterdam, Amsterdam, NLD; 3 Internal Medicine, Brookdale University Hospital Medical Center, Brooklyn, USA; 4 Internal Medicine, Smolensk State Medical University, Smolensk, RUS; 5 Psychiatry and Behavioral Sciences, Medical University-Sofia, Sofia, BGR; 6 Cardiology, Atlanta Veterans Affairs Medical Center, Decatur, USA

**Keywords:** covid-19, chest pain, google trends, pandemic, cardiovascular disease, acute myocardial infarction, acute coronary syndrome, sars-cov-2 (severe acute respiratory syndrome coronavirus-2)

## Abstract

Background

The coronavirus disease 2019 (COVID-19) pandemic has been linked to a myriad of cardiac symptoms and disorders. Reports also suggest decreased hospital visits by patients with known cardiovascular disorders.

Methodology

To better elucidate the public interest in the information regarding “chest pain” during the COVID-19 pandemic, we conducted a Google Trends analysis from March 2019 to March 2021 to compare the internet searches between pre-COVID era and during the pandemic with country-wise [the United States (US) versus the United Kingdom (UK) versus India] variation.

Results

We observed a significantly rising public interest in “chest pain” internet searches during the peak COVID-19 pandemic. Rising trends were most prominent in the UK, followed by USA and India. Our analysis noted a spike in the trend of “chest pain” search in early March in the UK and USA, whereas in March and June 2020 for India. This shows an important temporal association between the surge of COVID-19 cases and the search for “chest pain” online.

Conclusion

Google Trends analyses indicate rising public interest in chest pain during the pandemic months and the possible association between COVID-19 and chest pain. These findings warrant further research, especially with increasing reports suggesting contradictory reports of decreased hospital visits by patients with known cardiovascular diseases.

## Introduction

According to the World Health Organization, the current coronavirus disease 2019 (COVID-19) pandemic has infected around 116 million people and has led to approximately 2.6 million deaths worldwide. These patients present with a myriad of cardiopulmonary symptoms with chest pain taking the lead. Existing reports suggest a high burden of cardiac injury (19.7-27.8%) in patients with COVID-19 with a significantly high mortality rate [1,2]. The spectrum of cardiac involvement ranges from myocarditis, pericarditis, acute coronary syndrome, to stress cardiomyopathy, all of which can lead to chest pain. It is a known fact that cardiac symptoms like chest pain have a seasonal surge pattern, peaking around spring and winter [3]. The current COVID-19 pandemic started in the winter months of 2019 which is also the peak season for patients presenting with cardiac and pulmonary symptoms. Therefore, there can be an overlap between the seasonal surge in non-COVID-related cardiac symptoms and cardiac symptoms related to COVID infection. To better elucidate the public interest in the information on “chest pain” during the COVID-19 pandemic, we conducted a Google Trends search to analyze the internet searches for “chest pain” during March 2019 and March 2021 in the United States (US), United Kingdom (UK), and India where the burden of COVID-19 cases was substantially high throughout the pandemic period.

## Materials and methods

We conducted a Google Trends analysis using Google Trends (https://google.com/trends, Google LLC, Mountainview, CA, USA) from March 1, 2019 to March 13, 2021 to compare the internet searches for “chest pain” between the pre-COVID era versus during the pandemic with country-wise (US versus UK versus India) variation. Data were reported in relative [internet] search volumes (RSVs) with the search term “chest pain” and were compared between the three countries. RSVs are indicative of percentages relative to the peak search volume observed during a particular time period and scaled by the total search volume for each specific search term. Numbers do not represent absolute search volume, but instead the data are normalized and reported in this study on a scale from 0 to 100.

## Results

**Figure 1:**
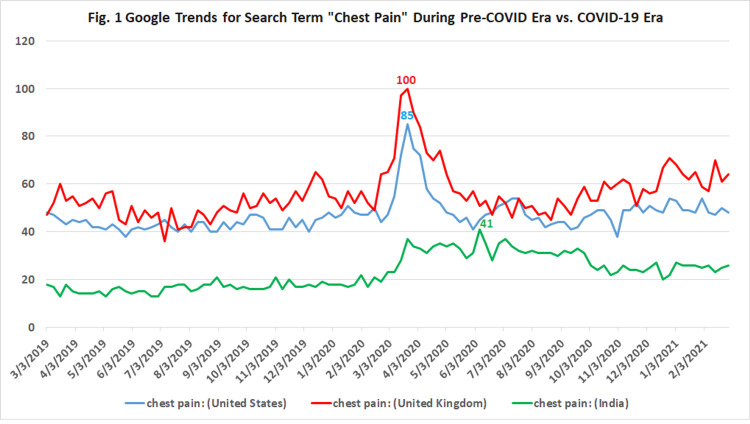
Google Trends analysis for search term “chest pain” during the pre-pandemic era in 2019 versus the pandemic months until March, 2021. Values are relative to the highest peak value of 100 indicating peak popularity for the keyword. A score of 0 denotes insufficient data for the term. Popularity in Google searches for terms containing "chest pain.” The left axis denotes interest in search terms.

As shown in Figure 1, we observed a significantly rising public interest in “chest pain” internet searches during the peak COVID-19 pandemic. Rising trends were most prominent in the UK followed by the US and India. Our analysis noted a spike in the trend of “chest pain” search in the early March in the UK and USA, whereas in March and June 2020 for India. This shows an important temporal association between the surge of COVID-19 cases and the search for “chest pain” online.

## Discussion

This Google Trends analysis is the first study to elucidate the public interest for “chest pain” during the COVID-19 pandemic compared to the pre-pandemic months in 2019. This analysis demonstrates rising trends in public interest in “chest pain” during the peak of the COVID-19 pandemic. Rising trends were most prominent in the UK followed by the US and India. Our analysis noted a spike in the trend of “chest pain” search in early March for the UK and US whereas in June 2020 for India. This trend gradually declined after June 2020 for all three countries. This hints towards an important temporal association between the surge of COVID-19 cases and the search for “chest pain” online. The utility of Google Trends in monitoring the outbreaks of this novel infection has already been reported before [4].

This remarkable surge of public interest in “chest pain” happened at the same time when elective and emergent procedures or admissions for cardiac diseases were reported to hit an all-time low across the globe [5]. This leads to the hypothesis that cardiac symptoms like chest pain might have happened more frequently during the pandemic among the general public; however, these symptoms were largely under-reported due to fear of seeking care from healthcare facilities or neglected by the general public being non-specific in nature [6]. It is evident that COVID-19 remains significantly underdiagnosed in many countries [7]. Therefore, the trends in increased searches for “chest pain” with simultaneously decreased hospital visits are concerning and should spark an alarm among healthcare professionals and policymakers, especially with new and more infectious strains are on the rise in various countries [8]. With the recent spike in cases caused by the new and possibly more infectious strains of the coronavirus, healthcare professionals and medical media personnel bear the responsibility of raising awareness and educating the general population about the potential deadly complication one may suffer from with underlying chest pain, especially without timely diagnosis and management. It is also important that patients should practice protective precautions, both in and out of the healthcare facilities to avoid being infected during hospital visits. However, the public needs to be encouraged to seek medical attention timely should they experience any degree of chest pain as it could be an early symptom of underlying severe cardiovascular conditions such as acute coronary syndrome or myocarditis or cardiac arrest.

There are some limitations associated with this analysis that need to be taken into consideration. These searches might be an under-representation of the cases from India largely due to the limited access to internet services in some areas. Furthermore, our analysis does not include searches performed on other search platforms other than “Google Search.” In this analysis, there are some speculative observations in the absence of granular patient-level data, that is, no detailed history of chest pain. However, our analysis serves well as a preliminary source of a broad-based population perspective on chest pain during the pre-pandemic era versus the pandemic months.

We encourage further research to find out the answers to the following questions.

Is this surge in the public interest of “chest pain” search mere anxiety-driven among patients with known cardiovascular disease during COVID-19 peaks or there remains a real underreported case burden of new-onset acute cardiac injury during the pandemic period?

Does the COVID-19 pandemic deter the general public from visiting healthcare facilities for medical attention and instead turn to look for their symptoms online?

What could be the possible reasons for the more prominent trends in the UK compared to other countries? Is the sociodemographic or genetic make-up of patients in the UK a predisposing factor for cardiac involvement and complications compared to patients from other countries?

Does high public interest in the “chest pain” search during this phase translate into worse cardiovascular outcomes in the short or long term?

Overall, the combination of the decline in healthcare visits for cardiac care and the surge in the searches for “chest pain” during the COVID-19 pandemic period sets off an alarm, especially amid reports suggesting rising cases by new more severe strains of coronavirus. This necessitates immediate strides in developing measures to encourage the public to seek timely medical advice and initiatives in promoting regular follow-up of high-risk patients with known cardiovascular diseases with their healthcare providers.

## Conclusions

In conclusion, Google Trends analyses indicate rising public interest in chest pain during the pandemic months and the possible association between COVID-19 and chest pain, which could be due to COVID-19 associated cardiac injury as reported recently. The trends were most prominent in the UK followed by the US and India. Google Trends may prove to be a handy tool in real-time regional monitoring of the COVID-19 pandemic, along with the associated public interest in cardiac symptoms, which could indicate underlying cardiac involvement with this contagious virus and help identify patients with undiagnosed cardiovascular diseases at an early stage.
